# Study of Sex Differences in Unmedicated Patients With Major Depressive Disorder by Using Resting State Brain Functional Magnetic Resonance Imaging

**DOI:** 10.3389/fnins.2022.814410

**Published:** 2022-03-31

**Authors:** Lan Mei, Yuting Wang, Chunyang Liu, Jingping Mou, Yizhi Yuan, Lihua Qiu, Qiyong Gong

**Affiliations:** ^1^Department of Radiology, The Second People’s Hospital of Yibin, Yibin, China; ^2^Department of Radiology, Southwest Medical University, Luzhou, China; ^3^Department of Radiology, Chengdu Medical College, Chengdu, China; ^4^Huaxi MR Research Center (HMRRC), Department of Radiology, West China Hospital of Sichuan University, Chengdu, China

**Keywords:** brain activity, major depressive disorder (MDD), functional magnetic brain imaging (fMRI), sex difference, amplitude of low-frequency fluctuation

## Abstract

Some important clinical characteristics of major depressive disorder (MDD) differ between sexes. We explored abnormal spontaneous neuronal activity in MDD patients using the amplitude of low-frequency fluctuation (ALFF) and its relationship to clinical manifestations in male and female patients, to seek the neural mechanisms underlying sex-related differences in depression. Twenty-five male MDD patients, 36 female MDD patients, and 25 male and 36 female matched healthy controls (HC) were included. The ALFF difference was investigated among four groups, and partial correlation analysis was used to explore a possible clinical relevance. The main effect results of sex difference were located in the bilateral caudate nucleus and posterior cingulate gyrus. *Post hoc* comparisons found that the male MDD patients showed decreased ALFF in the bilateral caudate nucleus and posterior cingulate gyrus when compared with female MDD patients/female HCs, and female MDD patients showed increased ALFF in the bilateral caudate nucleus and posterior cingulate gyrus when compared with male HCs. The average ALFF of the right caudate nucleus was positively correlated with illness duration in female MDD patients. Our results suggest that the sex-specific abnormal brain activity might be a potential pathomechanism of different symptoms in male and female MDD patients.

## Introduction

Major depressive disorder (MDD) is the commonest psychiatric disease. Male and female patients with depression show differences in important clinical features such as morbidity, suicide rate, and clinical symptoms. Women are more likely to show fatigue and drowsiness during the most severe episodes, while men with MDD are more likely to show more insomnia, impulsiveness, and psychomotor agitation and to have comorbid substance abuse disorders (Roeloffs et al., 2001; Khan et al., 2002; Marcus et al., 2005). Although women with MDD are more likely to attempt suicide, men are at higher risk of successful suicide (Oquendo et al., 2001). Although sex differences in clinical manifestations of MDD are obvious, the underlying neural mechanisms remain unclear.

Researchers have long been interested in exploring the neural mechanisms of sex differences in depression. Several neuroimaging studies reported sex differences in structural and functional brain imaging. In morphometric magnetic resonance imaging (MRI) studies, it was found that the gray matter volume (GMV) of the left cerebellum significantly increased and the GMV decreased selectively in the bilateral middle temporal gyrus and left ventral medial prefrontal gyrus, while the GMV decreased selectively in the left lingual gyrus and dorsal medial prefrontal gyrus in female patients (Yang et al., 2017). Ballmaier et al. found a sexual dimorphism in the prefrontal cortex volume of elderly depressive patients (Ballmaier et al., 2008). Another study reported sex-related volumetric deficits in the amygdala and anterior cingulate cortex in MDD patients (Hastings et al., 2004). Another study of resting electroencephalogram (EEG) revealed that the excitability dynamics of cortical networks differed as a result of sex differences (Jausovec and Jausovec, 2010) and reported that the EEG activities in stimulus and non-stimulus conditions were different between men and women (Wada et al., 1994). Furthermore, a study of resting-state functional MRI (fMRI) found sex differences in distributed abnormal resting-state brain activity in MDD patients, including parts of the frontoparietal network, auditory network, attention network, and cerebellum network (Yao et al., 2014). These different results in depression may be related to the differences in data analysis methods, sample size, participant demographics (duration of depression, age at onset, and duration of antidepression therapy), and the scanners used.

Functional MRI (fMRI) detects changes of blood oxygen level-dependent (BOLD) signal as an indirect measure of neuronal activity and is a powerful tool for non-invasive exploration of brain functions. The amplitude of low-frequency fluctuation (ALFF) reflects the activity of neurons in the resting state. This study set out to explore the sex differences of resting low-frequency amplitude in MDD and the correlation between these differences and clinical manifestations. Given that the antidepressant drugs might influence the brain function, only untreated MDD patients were included. In addition, the correlation between these different ALFF and clinical manifestations was also evaluated.

## Materials and Methods

### Participants

Twenty-five male and 36 female unmedicated MDD patients and 25 male and 36 female healthy controls (HC) were recruited in the current study. The MDD patients were recruited from inpatient and outpatient facilities at the Department of Psychiatry, West China Hospital of Sichuan University, and they were evaluated by experienced psychiatrists based on Diagnostic and Statistical Manual of Mental Disorders, Fourth Edition (DSM-IV) criteria of MDD. Inclusion criteria for patients were the following: (1) Whole patients were suffering from a major depressive episode and without any treatment for at least 2 years; (2) age ranged from 18 to 60 years; (3) the Hamilton Rating Scale for Depression (HAMD) 17-item (Hamilton, 1967) scores ≥ 18; (4) excluding other mental illnesses and neurological diseases such as bipolar disorder, intellectual disability, etc.; and (5) exclusion of women in gestation and lactation period. A total of 61 well-matched HC (25 men and 36 women) were collected by poster advertisements. Inclusion criteria for HC were the following: (1) All of HCs meet the non-patient version of the Structured Clinical Interview for DSM-IV (SCID). (2) ruling out of family history of mental illnesses or severe physical diseases. All subjects were right-handed. The study was approved by the Research Ethics Review Board of the West China Hospital of Sichuan University. After giving a complete description of the study, all the subjects gave written informed consent.

### Magnetic Resonance Imaging Scan Acquisitions

The imaging data were acquired using a 3T MR scanner (General Electric, Milwaukee, United States) with an eight-channel radio frequency coil. Whole participants were laid up in a head coil and filled with foam padding in case of head movement. Instructions of keeping motionless, eyes closed, and not to think systematically as far as possible were given to the subjects.

The structural images were obtained by T1-weighted three-dimensional spoiled gradient recalled echo (3D SPGR) sequences with the following scan parameters: repetition time (TR) = 8.5 ms, echo time (TE) = 3.4 ms, flip angle (FA) = 12°, number of slices = 156, slice thickness = 1 mm, voxel resolution = 1 × 1 × 1 mm^3^, data matrix = 256 × 256, field of view (FOV) = 240 mm × 240 mm. The resting-state functional images were captured by employing a gradient-echo echo-planar imaging (EPI) sequence with the following parameters: TR = 2,000 ms, TE = 30 ms, FA = 90°, slice thickness = 5 mm, slice gap = 0 mm, number of slices = 30, FOV = 240 × 240 mm, data 0matrix = 64 × 64, voxel resolution = 3.75 × 3.75 × 5 mm^3^, and acquisition time = 400 s (200 volumes).

### Data Preprocessing

The Data Processing Assistant for Resting-State fMRI (DPARSF) was used to perform standard preprocessing steps (Chao-Gan and Yu-Feng, 2010). The first five volumes were discarded to eliminate the effects of initial machine signal instability. Then, the remaining volumes were corrected for the acquisition time delay and head movement between slices. Translational or rotational motion thresholds of head movements were controlled within 2 mm or 2°. We calculated frame-wise displacement (FD) which reflected the head movement at every different time point by employing six displacements from the rigid body motion correction procedure (Power et al., 2012). The residual data were spatial normalized in the Montreal Neurological Institute (MNI) space, re-sampled with 3 × 3 × 3 mm^3^ resolution, and spatially smoothed using 8 mm full width at half maximum (FWHM) Gaussian kernel. To lessen the low-frequency drift, high-frequency noise, and cardiac noise, we used linear detrending and temporal filtering (0.01–0.08 Hz) for further ALFF analysis (Biswal et al., 1995).

### Statistical Analyses

One-way analysis of variance (ANOVA) was used to compare the distributions of age and education years between the whole groups. Illness duration and HAMD score were compared using two-sample *t*-test through SPSS 25.0 software among the MDD groups divided by sex. SPM8^1^ was employed to analyze the ALFF data. We put the complete voxel-based comparisons of the whole brain ALFF maps into a random-effects 2 (sex: man, woman) × 2 (group: MDD, HC) ANOVA model to figure out the impact of general differences in group, sex, and their interactions. We set the statistical significance threshold to *p* < 0.001 at the voxel level and a family wise error (FWE) corrected value of *p* < 0.05 at the cluster level using SPM to strictly control the false-positive rate. The regions that showed significantly different spontaneous neuronal activity between male and female subjects based on ALFF values were extracted as regions of interest (ROIs). SPSS 25.0 was used to perform further *post hoc t*-tests. *P* < 0.05 was considered statistically significant. We carried out a partial correlation analysis to find possible clinical correlation between the averaged ALFF values and clinical data (including the HAMD score and disease duration) in male and female MDD patients.

## Results

### Demographic and Clinical Characteristics of Participants

Twenty-five male and 36 female unmedicated MDD patients and 25 male and 36 female age- and education-matched HC completed the study. There was no sex difference of HAMD score between male and female MDD patients. However, the illness duration in the male MDD group was longer than in the female MDD group (Table 1).

**TABLE 1:** Demographic and clinical characteristics of all of the subjects.

Variables (mean ± SD)	Male MDD	Female MDD	Male HC	Female HC	p-value
Age (years)	36.4 ± 12.8	36.1 ± 10.7	34.9 ± 11.9	36.6 ± 11.8	0.842^a^
Education (years)	8.8 ± 3.2	8.6 ± 2.6	9.1 ± 3.1	8.3 ± 2.1	0.746^a^
HAMD score	22.3 ± 4.6	23.9 ± 4.5			0.188^b^
Illness duration (weeks)	101.2 ± 98.4	29.1 ± 42.5			0.003^b^

*SD, standard deviation; MDD, major depressive disorder; HC, healthy controls; HAMD, Hamilton Rating Scale for Depression; FD, frame-wise displacement.*

*^a^The p-values were obtained by one-way analysis of variance tests.*

*^b^The p-values were obtained by two-sample t-test.*

### The Sex Difference of Amplitude of Low-Frequency Fluctuation Among Four Groups

**FIGURE 1:**
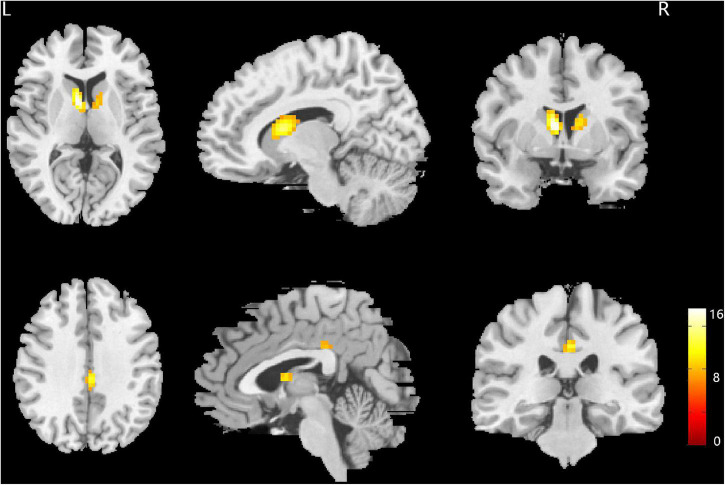
Brain regions showing the main effect result of amplitude of low-frequency fluctuation (ALFF) differences between male and female major depressive disorder (MDD) patients. *p* < 0.05, family wise error (FWE) corrected.

The main effect of sex difference was located in the bilateral caudate nucleus and posterior cingulate gyrus (FWE correction) (Figure 1 and Table 2). *Post hoc* comparisons found male MDD patients showed lower ALFF values than female HC/female MDD patients, female MDD patients showed higher ALFF values than male HC (detailed *post hoc* results in Table 3) (*p* < 0.05, Bonferroni corrected). There was no interaction between diagnosis and sex.

**TABLE 2:** Brain areas with significant amplitude of low-frequency fluctuation (ALFF) differences between the four groups of subjects.

Brain regions	Brodmann areas	MNI coordinate			Cluster size	F-value
		x	y	z		
Left caudate nucleus		−9	3	12	163	16.37
Right caudate nucleus		12	3	12	211	10.60
Posterior cingulate gyrus	23	0	−27	36	249	13.31

*MNI, Montreal Neurological Institute; x, y, z are the coordinates of primary peak locations in the MNI space; p < 0.05, corrected for FWE correction.*

**TABLE 3:** *Post hoc* comparisons of amplitude of low-frequency fluctuation (ALFF) among four groups.

		Left caudate nucleus		Right caudate nucleus		Posterior cingulate gyrus	
		Mean deviation	p-value	Mean deviation	p-value	Mean deviation	p-value
fMDD	mMDD	0.23524*	0.000	0.21211*	0.002	0.43503*	0.000
	fHC	–0.02036	1.000	–0.03906	1.000	0.18742	0.172
	mHC	0.20908*	0.000	0.21767*	0.001	0.44587*	0.000
mMDD	fMDD	−0.23524*	0.000	−0.21211*	0.002	−0.43503*	0.000
	fHC	−0.25560*	0.000	−0.25116*	0.000	−0.24762*	0.001
	mHC	–0.02616	1.000	0.00556	1.000	0.01084	1.000
fHC	fMDD	0.02036	1.000	0.03906	1.000	–0.18742	0.172
	mMDD	0.25560*	0.000	0.25116*	0.000	0.24762*	0.001
	mHC	0.22944*	0.000	0.25672*	0.000	0.25846*	0.001
mHC	fMDD	−0.20908*	0.000	−0.21767*	0.001	−0.44587*	0.000
	mMDD	0.02616	1.000	–0.00556	1.000	–0.01084	1.000
	fHC	−0.22944*	0.000	−0.25672*	0.000	−0.25846*	0.001

*fMDD, female major depressive disorder; mMDD, male major depressive disorder; fHC, female healthy controls; mHC, male healthy controls.*

*P < 0.05, corrected for Bonferroni correction.

### Explorative Correlation Analysis Between Amplitude of Low-Frequency Fluctuation Values and Clinical Data in Major Depressive Disorder

**FIGURE 2:**
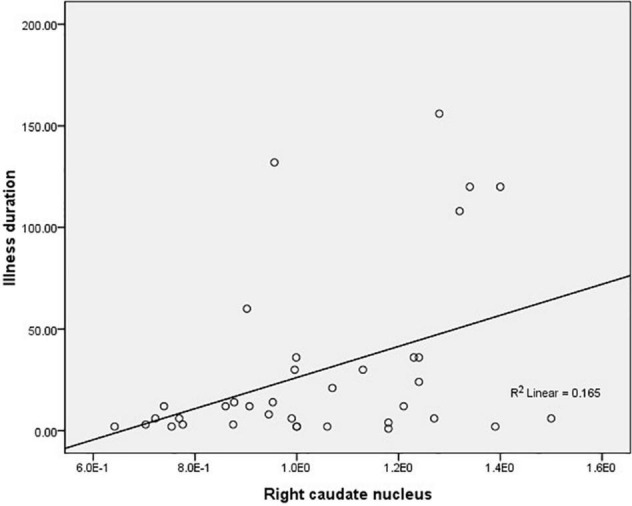
Correlations between the average amplitude of low-frequency fluctuation (ALFF) values of the right caudate nucleus and the illness duration in female major depressive disorder (MDD) patients.

The average ALFF values of the right caudate nucleus are positively correlated with the illness duration (*r* = 0.406, *p* = 0.01, corrected) in female MDD patients (Figure 2). There was no significant correlation in male MDD group.

## Discussion

In this study, we found that the main effect result of sex difference was located in bilateral caudate nucleus and posterior cingulate gyrus; on further *post hoc* comparisons, the male patients showed lower ALFF values in the bilateral caudate nucleus and posterior cingulate gyrus when compared with female MDD patients/female HC. We used ALFF approach in the current preliminary research, whose results are consistent with our proposal that sex differences might contribute to abnormal brain activity in MDD patients at resting state. Our findings may reflect the sex effect to clinical manifestations of MDD.

The caudate nucleus is important in information transfer between the thalamus and frontal lobe and plays an important role in regulating emotion, movement, and cognitive function. Studies have shown that there are sex differences in the volume and function of the caudate nucleus in normal people. The density of sex hormone receptor in the male caudate nucleus is higher than that in female caudate nucleus (Giedd et al., 2012), while the volume of the female caudate nucleus is larger than that of the male caudate nucleus (Sowell et al., 2002). Stevens and Hamann (2012) found that women showed greater activation than men in the left caudate head by using fMRI. The above researches support sex differences in the caudate nucleus. Consistent with the above studies, we also found that the female HCs showed higher ALFF values than male HCs in the bilateral caudate nucleus.

The caudate nucleus is the most prominent structure in the corpus striatum and plays an important role in the occurrence and development of depression (Kim et al., 2008). Ancelin et al. found smaller caudate nucleus and amygdalae in men with lifetime MDD (Ancelin et al., 2019). Some significant clinical features of MDD are in a sex-specific manner (e.g., more impulsiveness in men *vs*. more drowsiness in women) (Roeloffs et al., 2001; Khan et al., 2002; Marcus et al., 2005). Sex differences have been reported in impulse inhibition connected with the caudate nucleus of young adults, indicating different processing strategies (e.g., inhibiting inappropriate response in men *vs*. eliciting appropriate response in women) (Liu et al., 2012). This difference in the caudate nucleus reflects distinct biological correlates of MDD due to sex effects. It seems that estrogen has a neuroprotective effect on the striatum, implying decreased vulnerability in this region in women (Dluzen, 2000). Our results are consistent with these studies; thus, sex differences may cause baseline brain activity abnormalities in the bilateral caudate nucleus, leading in turn to decreased inhibition of prefrontal striatum neural circuit behavior in men with MDD, which may account for the increased impulsiveness and increased suicidal behaviors in men. In particular, the sex difference of ALFF values in the left caudate nucleus was more obvious but weakened in the right caudate nucleus of MDD patients compared with HC in our study, which implied that the left and right caudate nucleus might play different roles in MDD. However, these conclusions are tentative, and how the sex-specific mechanism affects the emergence or development of depression is unknown; further longitudinal studies are needed to verify this speculation.

Further analysis showed that the mean ALFF values in the bilateral caudate nucleus was positively correlated with illness duration in female MDD patients. The duration of depressive disorders is associated with clinical symptoms (Yao et al., 2014). Based on our results and previous findings, we speculated that the higher ALFF in female MDD patients may be a compensatory mechanism due to the illness duration being relatively short. The neuroprotective effect of estrogen on the striatum in female MDD patients may lead to the activation of the bilateral caudate nucleus to regulate negative emotions in female MDD patients before behavioral features manifest. Although changes in ALFF values in the bilateral caudate nuclei in female MDD patients can reflect the duration of the disease at an earlier stage, it is not clear how long this correlation will last. The interpretation is limited by the cross-sectional research design.

Amplitude of low-frequency fluctuation (ALFF) in the posterior cingulate gyrus in male MDD patients decreased compared with female MDD patients, which was more severe than that in HC. The cingulate cortex shows sex differences in structure and function. A morphological study on MRI found women had relatively larger cingulate GMV than men overall (Mann et al., 2011). The posterior cingulate gyrus is a key region in the default-mode network (DMN) (Raichle, 2015). The DMN is unique in showing a strong activity reduction during cognitive task performance (Fox and Raichle, 2007; Lee et al., 2013), which is essential for successful engagement in overt cognitive processing memory encoding (Gusnard et al., 2001; Daselaar et al., 2004). The posterior cingulate gyrus plays a central role in supporting internally directed cognition (Buckner et al., 2008; Raichle, 2015) and regulating the focus of attention (Hahn et al., 2007). It was reported in functional connectivity (FC) studies that FC in the DMN was stronger within the posterior cingulate cortex/precuneus and bilateral medial prefrontal cortex of women (Bluhm et al., 2008) as well as stronger in the intra-network, while it is stronger in the inter-network of men (Allen et al., 2011). Previous studies found potential sex differences in social cognition mediated by the DMN (Zhang et al., 2018). Sex differences were also found in the FC strength between default state network and frontoparietal network during cognitive control in self-referential processing context (Alarcón et al., 2018). There are sex differences in emotion recognition, and it has been speculated that the two sexes may differ in the brain areas which are activated during emotion recognition of facial expressions (Lee et al., 2002). There are sex differences in the degree of reaction in the posterior cingulate gyrus responses to emotional stimuli (Hofer et al., 2006), and male MDD patients show more impairment than women in this task (Kornstein et al., 2000). The abnormal spontaneous neural activity which was reflected by ALFF abnormalities in the posterior cingulate gyrus may contribute to differences in cognitive and attention patterns between male and female MDD patients. This might be related to the different clinical characteristics of the sexes.

Several issues should be noted. Firstly, the result could not rule out different spontaneous cognitive processing between the sexes during resting state scanning. Secondly, this study was cross-sectional in design and cannot address how ALFF values would change as the illness develops. Longitudinal follow-ups will help to identify these dynamic processes as well as the mechanisms of cognitive decline. Finally, the illness duration of the male MDD group was longer than that of the female MDD group. Thus, the sex difference of ALFF could not exclude the influence of illness duration.

In summary, this study demonstrated that the sex differences in clinical manifestations of male and female MDD patients may have a brain functional basis. These different sex-related neural defect patterns strongly suggest that the developmental and pathophysiological mechanisms of MDD differ in male and female patients, which might lead to sex differences in the clinical manifestations of MDD. In addition, they may be of significance for the development of sex-specific and more effective MDD targeted therapy.

## Data Availability Statement

The original contributions presented in the study are included in the article/supplementary material, further inquiries can be directed to the corresponding author.

## Ethics Statement

The studies involving human participants were reviewed and approved by the Research Ethics Review Board of the West China Hospital of Sichuan University. The patients/participants provided their written informed consent to participate in this study.

## Author Contributions

LM wrote the manuscript and contributed to analysis of the data. YW contributed to the manuscript writing. LQ, CL, and JM contributed to management and analysis of the imaging data. YY conducted the study. QG and LQ designed the study. All authors contributed to the manuscript and approved its final version.

## Conflict of Interest

The authors declare that the research was conducted in the absence of any commercial or financial relationships that could be construed as a potential conflict of interest.

## Publisher’s Note

All claims expressed in this article are solely those of the authors and do not necessarily represent those of their affiliated organizations, or those of the publisher, the editors and the reviewers. Any product that may be evaluated in this article, or claim that may be made by its manufacturer, is not guaranteed or endorsed by the publisher.

## Footnotes

1
http://www.fil.ion.ucl.ac.uk/spm/software/spm8/
